# Simultaneous Presentation of Gastric and Orbital Mucosa‐Associated Lymphoid Tissue Lymphoma: A Case Report

**DOI:** 10.1002/ccr3.9712

**Published:** 2024-12-10

**Authors:** Elahe Mirzaee, Maryam Garousi, Shima Jafari, Sepideh Soltani, Nasser Rakhshani, Navid Abdi, Pedram Fadavi

**Affiliations:** ^1^ Department of Radiation Oncology, School of Medicine Iran University of Medical Sciences Tehran Iran; ^2^ Gastrointestinal and Liver Diseases Research Center, Firoozgar Hospital Iran University of Medical Sciences Tehran Iran; ^3^ Department of Pathology, School of Medicine Iran University of Medical Sciences Tehran Iran

**Keywords:** case report, gastric, maltoma, orbital

## Abstract

Mucosa‐associated lymphoid tissue lymphoma or MALToma occurs in 8% of B‐cell non‐Hodgkin lymphoma according to the latest WHO classification. The most involved site of MALToma is stomach. We describe a rare case of concurrent gastric and orbital mucosa‐associated lymphoid tissue lymphoma (MALToma) of a female who presented with progressive proptosis and abdominal pain. We performed six courses of chemotherapy (Rituximab) and gastric and orbital radiation with a dose of 34 Gy/20 Fr and 30 Gy/20 Fr, respectively. This case underscores the efficacy of a multimodal treatment strategy in achieving successful outcomes.

**Summary**

- Primary concurrent orbital and gastric MALToma is a rare clinical entity.
- It can be treated with multimodality treatment after staging.
- The results obtained from this case report will help treat this uncommon condition.

## 1 Introduction

Mucosa‐associated lymphoid tissue lymphoma or MALToma occurs in 8% of B‐cell non‐Hodgkin lymphoma according to the latest WHO classification [1].

The most involved site of MALToma is stomach but affects other sites such as ocular adnexal, salivary gland, lung, colorectal, and several unusual sites [2]. Unlike children with a progressive course, it tends to be indolent in adults [3].

Chronic infection or autoimmune conditions can activate the immune system, potentially leading to the development of MALToma. Evidence supports a robust correlation between *Helicobacter pylori* and MALToma. A treatment option for gastric MALToma is *H. pylori* elimination. Chlamydophilia Psittaci is another infection implicated in ocular MALToma. Patients with gastric MALToma are often asymptomatic and discovered with screening esophagogastroduodenoscopy [2].

Ocular adnexal lymphoma can initially involve eye structure or be secondary to similar lymphoma in another site. The most common primary ocular adnexal lymphoma type is a low‐grade type such as MALToma. In up to one‐third of cases, patients with primary ocular lymphoma progress to systemic lymphoma, but only 5% of patients with non‐Hodgkin lymphoma develop ocular adnexal disease [4].

Most ocular adnexal lymphoma cases are asymptomatic, but the location of the lesion is important and may present with pain, diplopia, eyelid swelling, proptosis, or double vision [4].

This case report presents one case with concurrent involvement of stomach and ocular MALToma.

## 2 Case History and Examination

A 63‐year‐old woman, experiencing progressive proptosis and enduring abdominal pain for 6 months, was referred to Hafte Tir Hospital in Tehran, Iran. There was no disease in the individual or family history. Ophthalmic examination revealed a visual acuity of 10/10 in both the eyes. The ocular pressure of the right and left eye was 22 and 18 mm Hg, respectively. She had a mobile and painless mass in her right and left upper lid. The optic disk and fundus examination were normal in both eyes. No regional lymph node enlargement was observed.

### 2.1 Differential Diagnosis, Investigations, and Treatment

**FIGURE 1:**
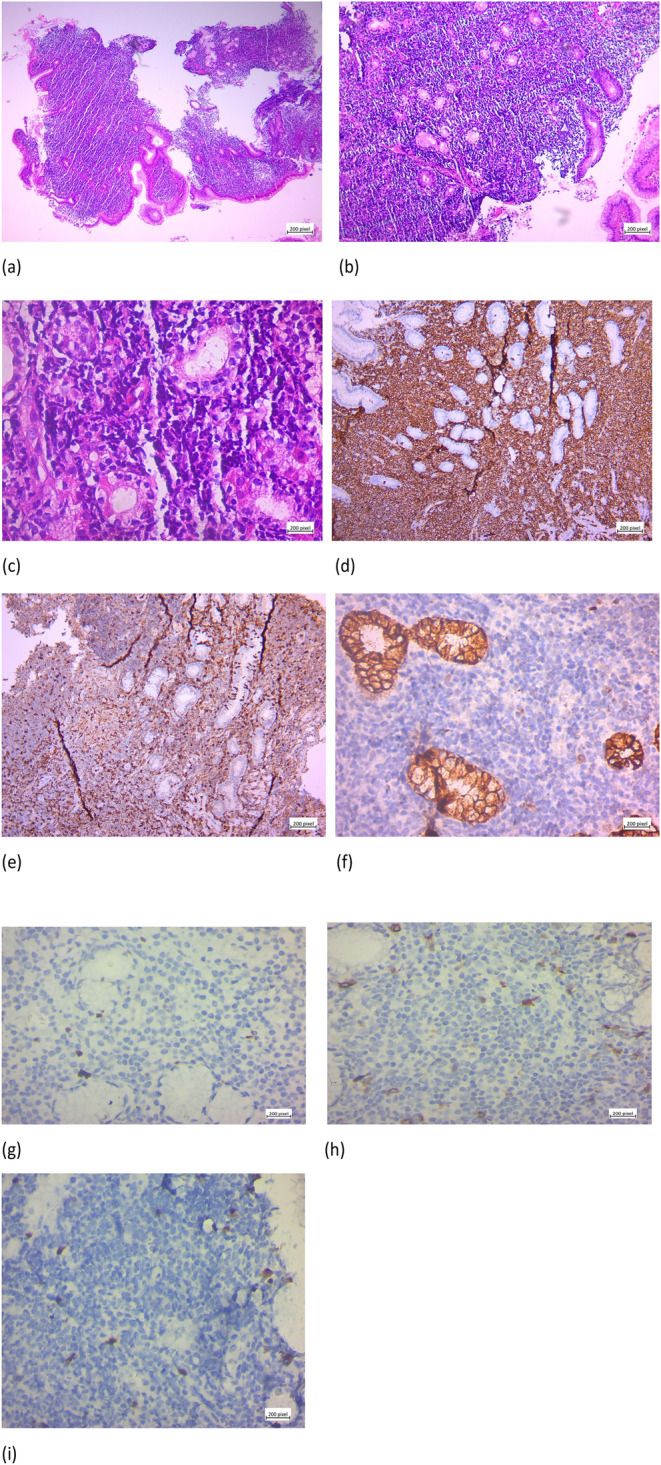
(a) H&E ×4: Sheets of dense small neoplastic lymphocytes infiltrating gastric mucosa. (b) H&E ×10: Sheets of neoplastic cells are seen, and lymphoepithelial lesions become more apparent. (c) H&E ×40: Lymphoepithelial lesions as well as lamina propria infiltration by centrocyte‐like cells mixed by monocytoid lymphocytes and some plasmacytic differentiation. (d) H&E ×10: Neoplastic lymphocytes express diffusely CD20 marker in IHC staining. (e) H&E ×10: CD43 is expressed by most of neoplastic lymphocytes in IHC evaluation. (f) H&E ×40: Ki‐67 proliferative index is low (5%) in this low‐grade lymphoma. (g) H&E ×40: Cyclin D1 is negative in neoplastic lymphoid cells. (h) H&E ×40: CD5 is negative in tumoral cells. (i) H&E ×40: CD10 is negative in tumoral cells.

The laboratory tests were normal, including cell blood counts (WBC: 4.5 10^3^/μL [4–10 × 10^9^/L], Hb: 12 g/dL [12–16 g/dL], Plt: 256 10^3^/mm^3^ [150–450 × 10^9^/L]) and the liver tests AST: 18 U/L [10–40 U/L], ALT: 10 U/L [10–40 U/L], ALP: 125 IU/L [30–120 U/L], total bilirubin: 1.06 mg/dL [0.3–1.0 mg/dL] and kidney (urea: 40 mg/dL [12–45 mg/dL], creatinine: 0.8 mg/dL [0.8–1.3 mg/dL]), thyroid function test (TSH: 3.5 mIU/L [0.5–5 mIU/L]), lactate dehydrogenase (LDH: 200 U/L [60–260 U/L]), serum beta‐2 microglobulin: 5 mg/L [1.5–3 mg/L], hepatitis B surface antigen (HBS Ag: negative), and antinuclear antibody was normal. Because of gastrointestinal symptoms, the patient was evaluated by endoscopy. It showed a large subepithelial lesion with multiple ulcerations in the cardia and fundus and lesser curvature. A biopsy was done. It showed severe gastritis and negative *H. pylori* . The patient was evaluated by endosonography‐guided fine‐needle aspiration biopsy. A large heterogenous lesion (50*20 mm) in the fundus and cardia and proximal of lesser curvature was seen. Multiple lymph nodes were detected. Pathology examination showed ulcerated gastric mucosa with atypical dense small lymphoid infiltration (Figure 1).

The immunohistochemical (IHC) study was positive for CD20, CD3, and CD43 and negative for CK, cyclin D1, CD5, and CD10. KI67 was 5% (Figure 1).

*H. pylori* was negative. The pathological findings were consistent with low‐grade gastric Mucosa‐associated lymphoid tissue (MALT) B‐cell lymphoma.

**FIGURE 2:**
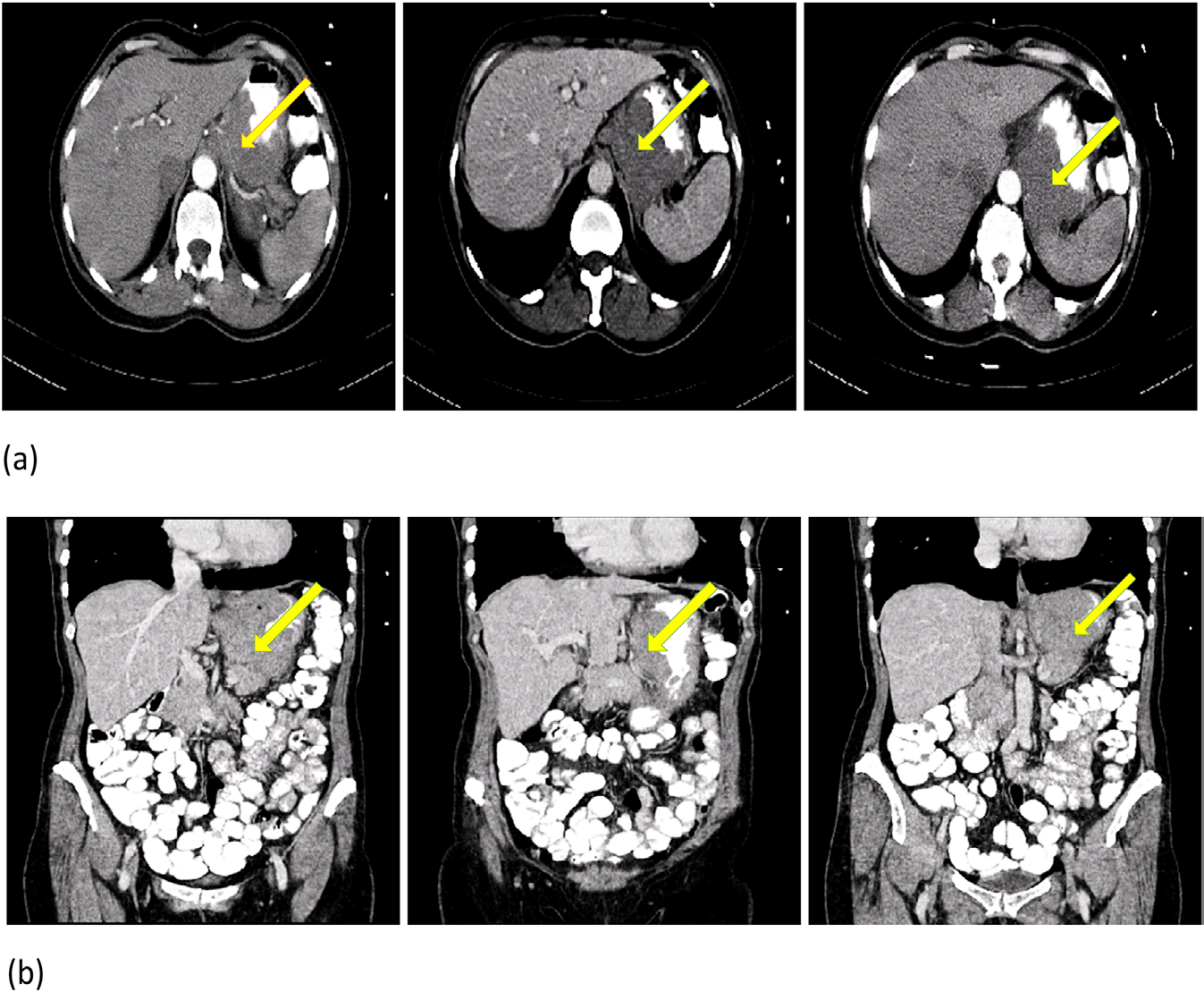
CT scan of the abdomen and the pelvis with and without injected and oral contrast after first admission. The yellow arrow shows a mass in the gastric fundus with extension to lesser curvature. (Panel A: Axial view. Panel B: Coronal view).

A computed tomography scan (CT scan) of the chest, the abdomen, and the pelvis with and without contrast injection and oral was performed. A mass was observed in the gastric fundus, extending to lesser curvature (Figure 2).

The patient received six courses of chemotherapy (500 mg Rituximab every 28 days). She responded well, and proptosis and abdominal pain were resolved.

### 2.2 Outcome and Follow‐Up

**FIGURE 3:**
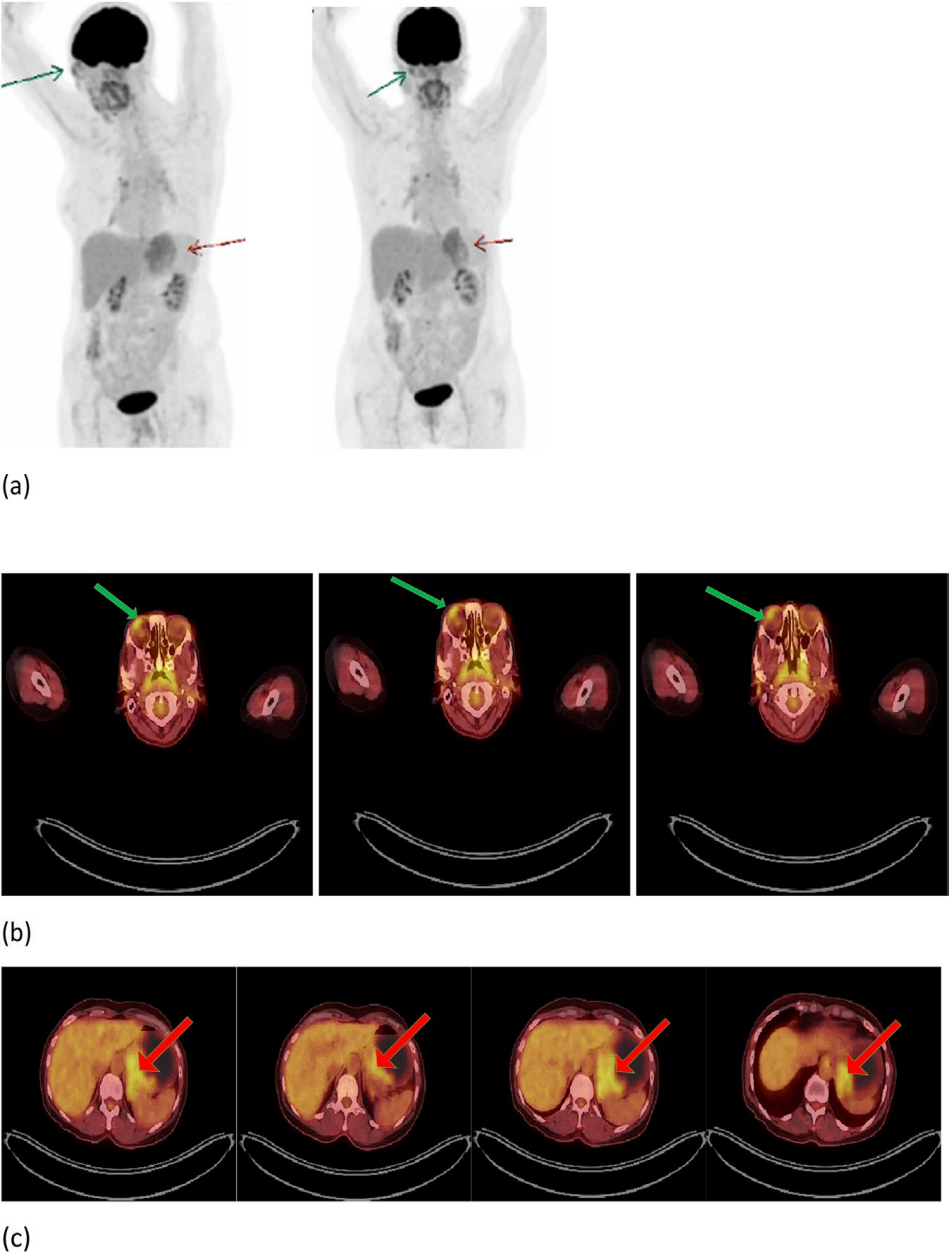
Whole‐body FDG‐PET CT scan after 2 months of chemotherapy. (Panel A): Maximum intensity projection (MIP) PET imaging: The green‐yellow: Orbital lesion. The red‐yellow: The gastric lesion. (Panel B): The green‐yellow show increased metabolic activity in the supralateral aspect of the right orbit. (Panel C): The red‐yellow shows hypermetabolic gastric wall thickening in the fundus and cardia with extension to the lesser curvature, consistent with the residual metabolic activity.

After 2 months, an FDG‐PET CT scan was done. Hypermetabolic gastric wall thickening in the fundus and cardia with extension to the lesser curvature, consistent with the residual metabolic activity with a Deauville score of 4 and increased metabolic activity in the supralateral aspect of the right orbit, were seen. Increased FDG uptake in the nasopharynx is secondary to an inflammatory process (Figure 3).

Because of residual disease in gastric and orbital, she underwent external gastric and orbital radiation with a dose of 34 Gy/20 Fr and 30 Gy/20 Fr, respectively.

**FIGURE 4:**
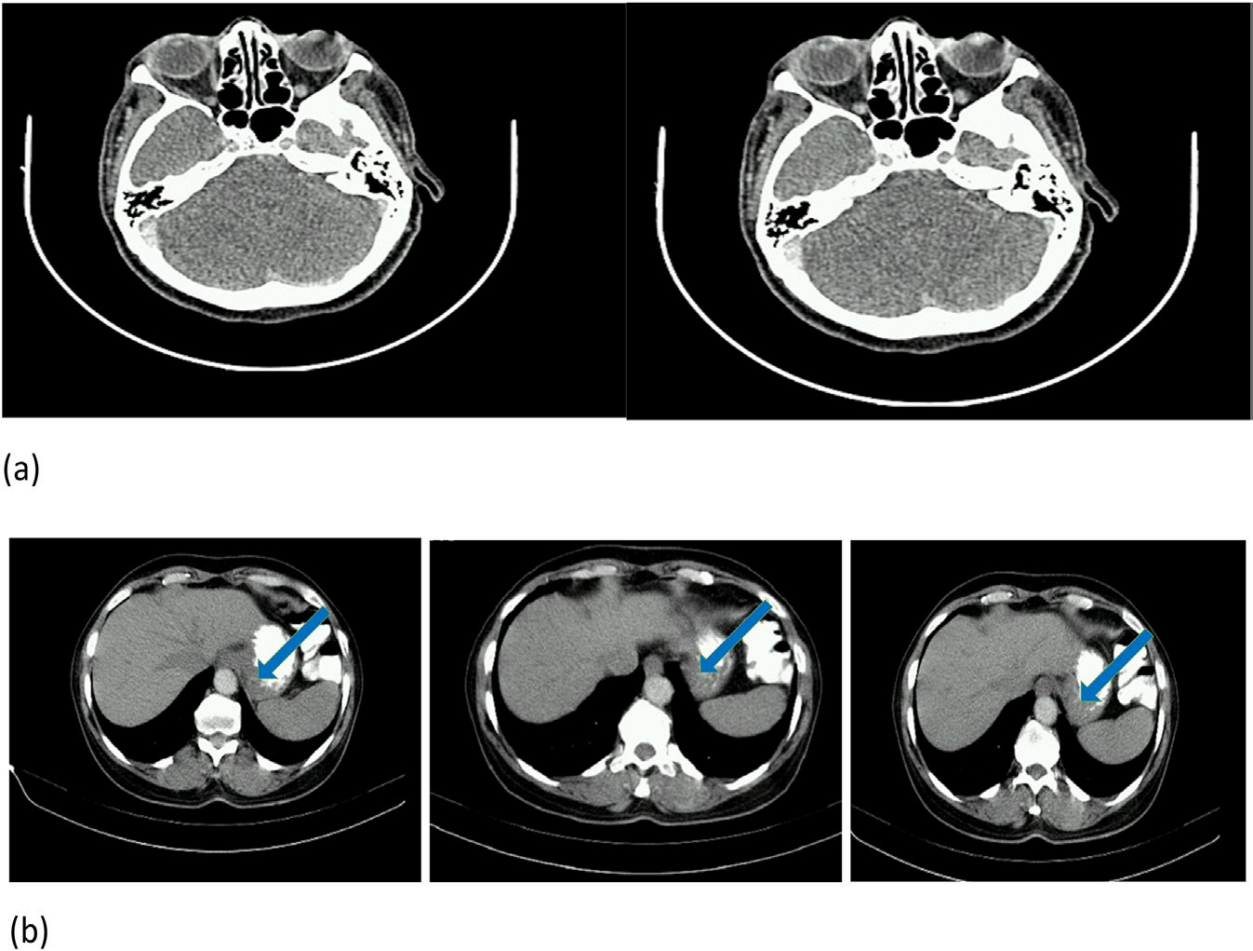
CT scan of the head and the abdomen with and without injected and oral contrast after 3 months of treatment. (A) The right orbit without lesion. (B) The blue yellow shows a slight wall thickening in fundus and cardia in the gastric.

3 months after the completion of all treatments, a follow‐up CT scan was performed. It revealed no lesion in the right orbit and wall thickening in the fundus and cardia in the gastric (Figure 4).

## 3 Discussion

The stomach is the most common site of extranodal lymphoma involvement. MALT contributes 50% of gastric lymphoma and is characterized by indolent behavior; however, approximately 10% of patients present with Stage 3 and 4 diseases. The most frequent sites of metastasis are lymph nodes and other organs like the lung, liver, and bone marrow [5].

In addition to the stomach, the other common primary sites of MALT lymphoma are salivary glands, skin, orbits, conjunctiva, lungs, thyroid, upper airway, breast, and liver [6]. Bone marrow is involved in 20% of cases, which causes a worse prognosis [6].

MALToma tends to be localized, but multi‐organ involvement is not uncommon. A retrospective study assessed 140 patients with MALToma. A total of 61 patients are included in the primary stomach category, and 15 patients had multi‐organ involvement, 6 in the extra gastrointestinal system including lungs, parotid, bladder, kidney, and bone marrow. Extra gastric primary MALTomas were more likely to involve other organs rather than gastric MALToma. Multifocal gastric MALToma was related to t(11;18) and (q21;q21) [7].

A retrospective analysis of 35 patients with MALToma (24 patients with gastric primary) showed 37% had lymph node involvement; however, only one of them had a gastric origin. 23% had concurrent multi‐organ involvement (1 parotid and lacrimal gland, 1 conjunctiva and hypopharynx, 1 conjunctiva and skin, 1 lacrimal gland and lung, 1 stomach and colon, 1 stomach and lung, 2 bilateral parotids) [8].

Based on these studies, nearly a quarter of MALToma have multi‐organ dissemination, which illustrates the importance of accurate staging assessment in these patients.

Staging workup for gastric MALToma includes endoscopy, biopsy, laboratory tests (LDH, CBC, biochemical study, B2 microglobulin), CT scan of thorax, abdomen, and pelvic, bone marrow aspiration, and biopsy [9].

Despite the high sensitivity of FDG/PET in aggressive tumors like large B‐cell lymphoma, its sensitivity in indolent tumors is low. The sensitivity of FDG/PET in extranodal lymphoma varies between 0% and 81%, according to different studies.

In a study, the sensitivity of PET/CT in 37 biopsy‐proven MZL patients was reported to be 59.5%. So, PET/CT could be a helpful modality in diagnosis and follow‐up [2]. In our case, there was uptake in both orbit and stomach. CXCR4 is expressed by MZL cells. Recently, CXCR4 PET/CT has been evaluated in MZL, and the results are promising [10]. Imaging features may help diagnose unusual sites of marginal zone lymphoma. Differential diagnoses of orbital lymphoma consist of metastasis, bilateral thyroid orbitopathy, lacrimal gland carcinoma, and IgG4‐related orbital pseudotumor. In imaging, MALToma appears as a mass in the upper outer part of the orbit, which may involve the lacrimal gland or rectus muscle. MRI is superior to a CT scan since it shows soft tissue more accurately [11]. Histopathology of MALToma illustrates lymphocyte infiltration in lamina properia in combination with gland destruction. Unlike DLBCL, which contains large cells, small cells are the prominent cell types in this histology [12]. IHC features of MALToma include diffuse positive CD20 and negative PAX‐5 [13].

MALToma in different sites has different prognosis and natural history. In a study including 167 MALToma patients with different primary sites who underwent radiation, the overall survival rate was 87%, and the best outcome was related to stomach and thyroid primaries. MALToma usually remains localized for a long time, but other site involvement can occur. In 20% of patients, bone marrow involvement is expected. Bone marrow and lymph node involvement is correlated with worse prognosis, but not those with multiple site involvement [6].

Treatment of gastric MALToma is based on the stage of the disease. Localized *H. pylori* disease should be treated with antibiotic therapy for 10–14 days with a combination of PPI, clarithromycin, and amoxicillin or metronidazole. Levofloxacin is used when the first‐line treatment fails. Antibiotic therapy causes 70%–100% long‐term remission. There is no evidence of additional treatment in patients who respond to antibiotic therapy [14].

Gastric and perigastric radiation with the dose of 30 Gy leads to 93%–100% remission. Chemotherapy or rituximab is used in advanced disease. Combination therapy with rituximab and chemotherapy, including alkylating agents or purine nucleoside analogs, is superior to single therapy [15]. In our case, there was a dramatic response to rituximab in both orbit and gastric disease, but consolidation radiotherapy was administered according to remnant disease in PET/CT.

## 4 Conclusion

This case is about a patient with gastric and orbital mucosa‐associated lymphoid tissue lymphoma. Following chemotherapy and radiotherapy, the patient responded well.

The results of this case will help in relation to similar cases.

## Author Contributions

**Elahe Mirzaee:** writing – original draft. **Maryam Garousi:** writing – review and editing. **Shima Jafari:** writing – review and editing. **Sepideh Soltani:** writing – review and editing. **Nasser Rakhshani:** data curation, investigation. **Navid Abdi:** data curation, investigation. **Pedram Fadavi:** writing – review and editing.

## Ethics Statement

We obtained a written statement of informed consent from the patient for the publication of case details and the use of images. The case discussed in this manuscript does not include patient‐identifying information, nor does it report a new study that required IRB approval.

## Conflicts of Interest

The authors declare no conflicts of interest.

## Data Availability

The data that support the findings of this study are available from the corresponding author upon reasonable request.
